# Relation among Mercury, Selenium, and Biomarkers of Oxidative Stress in Northern Pike (*Esox lucius*)

**DOI:** 10.3390/toxics11030244

**Published:** 2023-03-05

**Authors:** Jason T. Magnuson, Mark B. Sandheinrich

**Affiliations:** Department of Biology and River Studies Center, University of Wisconsin-La Crosse, La Crosse, WI 54601, USA

**Keywords:** Hg:Se molar ratios, northern pike, oxidative stress, gene expression, field collected

## Abstract

Mercury (Hg) is a toxic environmental contaminant associated with oxidative stress in freshwater fish. A known antagonist to Hg, selenium (Se), may reduce the toxic effects of Hg. In this study, the relation among Se, methylmercury (MeHg), inorganic mercury (IHg), total mercury (THg), and the expression of biomarkers of oxidative stress and metal regulation in livers of northern pike were examined. Livers from northern pike were collected from 12 lakes in Isle Royale National Park, Pictured Rocks National Lakeshore, Sleeping Bear Dunes National Lakeshore, and Voyageurs National Park. The concentrations of MeHg, THg, and Se were measured in liver tissue, and the expression of superoxide dismutase (*sod*), catalase (*cat*), glutathione s-transferase (*gst*), and metallothionein (*mt*) was assessed. There was a positive relationship between the concentrations of THg and Se, with a Hg:Se molar ratio less than one in all livers examined. There was no significant relation between *sod*, *cat*, *gst*, or *mt* expression and Hg:Se molar ratios. *cat* and *sod* expression were significantly related to increases in percent MeHg, relative to THg; however, *gst* and *mt* expression were not significantly altered. This suggests that incorporating biomarkers containing Se may be a better indicator than non-selenium-containing proteins of assessing the long-term effect of Hg and the interactions between Hg and Se in the livers of fish, such as northern pike, especially when molar concentrations of Se are greater than Hg.

## 1. Introduction

Mercury (Hg) is a toxic compound that exists primarily in three common forms in the environment: elemental mercury (Hg°), inorganic mercury (Hg^2+^), and methylmercury (MeHg) [1,2]. In addition to natural sources, prominent anthropogenic sources of mercury include chlor-alki plants [3] and atmospheric deposition from coal and artisanal mining [4,5,6]. Highly toxic MeHg is produced primarily by sulfate-reducing bacteria in aquatic environments [7]. Methylmercury bioaccumulates and biomagnifies in aquatic organisms, with exposure to MeHg from water, sediment, and food [7,8]. While fish accumulate only small amounts of Hg^2+^ [9], MeHg is readily bioavailable to fish. As trophic levels increase, the proportion of total Hg present in organisms as MeHg also increases and represents more than 90% of the mercury present in fish [10,11].

Exposure to environmentally relevant concentrations of MeHg impair growth, development, and reproduction in various vertebrates including birds [12,13], mammals [14,15], reptiles [16,17], and fish [1,7,18,19]. At the cellular level, MeHg causes oxidative stress through the production of reactive oxygen species (ROS), which in turn, causes lipid peroxidation and cell death [20,21,22]. Bioaccumulation and toxicity of MeHg may be ameliorated by other factors, such as selenium, which alters uptake and has a role in redox defense [23].

Selenium (Se) is a known antagonist to Hg, as first demonstrated by Parízek and Ostádalová (1967) [24], and supported by subsequent investigations [2,23,25,26,27,28,29]. Selenium is a naturally occurring element that was first noted for its toxicity, but subsequently acknowledged as an essential trace element required for the synthesis of more than 25 proteins [30,31]. This trace element can be found in a variety of forms, and its chemical form impacts its interaction with Hg [32]. In aquatic environments, Se is commonly found as water-soluble, inorganic selenite (SeO_3_^2−^) and selenate (SeO_4_^2−^), and as selenocysteine (SeCys) and selenomethionine (SeMet) [33]. Selenocysteine and SeMet are predominate Se-containing amino acids found in food [23], and reduce MeHg toxicity in fish at a greater rate than dietary selenate [25], which is taken up more slowly in aquatic organisms [34]. 

Although the binding of Se to Hg is important in reducing Hg toxicity, available Se is reduced by Hg [2], and subsequently considered inaccessible for further protein synthesis [23]. Therefore, it is important to consider molar ratios of Hg:Se, as opposed to just Hg concentrations, when assessing Hg toxicity to organisms. The protective effect of Se occurs when a molar ratio of 1:1 Se to Hg is approached or exceeded [23]. In this 1:1 stoichiometric state, Se decreases or prevents oxidative damage caused by Hg, which at toxic concentrations ultimately changes selenoenzyme activities and levels [28]. Since the bioaccumulation of high amounts of Se may result in selenosis, a narrow margin between safe and toxic exposure to Se exists [35]. A number of compounds and enzymes may mitigate oxidative stress associated with MeHg. These include glutathione (gsh), selenoproteins (e.g., iodothyronine deiodinases (dio), thioredoxin reductases (tr), and glutathione peroxidases (gpx)), superoxide dismutase (sod), catalase (cat), metallothionein (mt), and glutathione s-transferase (gst). However, a lack of clarity in the protective effects of Se against Hg toxicity in fish still remains, which may include altered bioavailability, binding affinity, and influence of overall fish health [36]. Differences in effects observed may also be due to the exposure route and duration in laboratory-based experiments compared to those from field-collected samples.

Chronic exposure to low levels of MeHg poses a threat to the health of wild fish populations. While it is understood that Se can help ameliorate the toxicity of Hg, it is still not understood if examining Hg:Se molar ratios is appropriate for addressing oxidative stress in freshwater fish. Therefore, the objective of this study was to determine the relation among Hg:Se molar ratios in the livers of wild caught northern pike (*Esox lucius*) and altered expression of *sod*, *cat*, *gst*, and *mt* when Se was in excess of Hg.

## 2. Materials and Methods

### 2.1. Experimental Design

Northern pike were collected from lakes in Isle Royale National Park (ISRO) (lakes Angleworm, Richie, Sargent), Pictured Rocks National Lakeshore (PIRO) (lakes Beaver, Grand Sable, Miners), Sleeping Bear Dunes National Lakeshore (SLBE) (lakes Bass (Benzie County), Bass (Leelanau County), Round), and Voyageurs National Park (VOYA) (lakes Brown, Peary, Ryan, Sand Point) as part of a project investigating mercury contamination of fish in national park units of the western Great Lakes region [37]. Northern pike were collected by angling and with gill nets in May 2011 and May 2012. After capture, fish were euthanized with a sharp blow to the head and cervical dislocation. A small plug of liver was removed with a flame-sterilized cork borer and preserved in RNAlater ^®^ (Life Technologies, Carlsbad, CA, USA). Samples were refrigerated for 24 h and then stored at −20 °C until analysis for gene expression. The remaining portion of liver was placed in a zip-closed plastic bag and frozen in a conventional freezer until analysis for mercury, methylmercury, and total selenium. 

### 2.2. Measures of Gene Expression

Livers (n = 94) were homogenized with polypropylene pestles (USA Scientific, Ocala, FL, USA) and RNA was isolated with an RNeasy Mini Kit (Qiagen, Valencia, CA), following the manufacturer’s instructions. Complementary DNA (cDNA) was synthesized from 1 µg of DNase treated RNA with iScript Reverse Transcription Supermix for RT-qPCR (Bio-Rad, Hercules, CA, USA). The concentrations of cDNA were determined by spectrometry (Nanodrop^®^, Thermo Fisher Scientific, Waltham, MA, USA). Primer pairs (Table 1) were designed with PrimerQuest software from Integrated DNA Technologies, following the manufacturer’s instructions. A gradient PCR was conducted for all target primers to denote optimal annealing temperature and determined that all products contained a single band. RT-PCR was run on a T100™ Thermal Cycler (Bio-Rad) using the following protocol: a 4 min activation and denaturing step at 95 °C, followed by 30 cycles of a 30 s denaturing step at 95 °C, a 30 s annealing step at 56 °C, and a 30 s synthesis step at 72 °C. A 10 µL qPCR reaction was performed with designed primers using SsoAdvanced Universal SYBR Green Supermix Kit (Bio-Rad), following the manufacturer’s instructions. mRNA expression was normalized to the internal reference primer, ubiquitin, as this gene did not significantly differ between fish sample or location. Each qPCR run was conducted in triplicate using the CFX Manager software, with a ∆∆Ct method used to assess changes in gene expression. 

### 2.3. Mercury Determination

Methylmercury was determined by digesting individual liver samples at 60 °C for 12 h in a 22 mL Teflon vial with 7 mL of 4.5 M nitric acid (adapted from [38]). A 100 µL aliquot of digestate, 100 µL of 4.5 M potassium hydroxide, 50 µL of 1% (*w*/*v*) sodium tetraethylborate, 400 µL of 2 M acetate buffer, and deionized water were added to fill a 40 mL borosilicate glass autosampler vial. The vials were analyzed by gas chromatography separation and cold-vapor atomic fluorescence spectrophotometry (CVAFS) [39,40] using a Brooks Rand MERX-M analyzer. Total mercury (THg) was determined from the same digestate used for MeHg analyses. To further oxidize each digestate, 2 mL of 0.2 bromine monochloride was added to the vial, followed by 12 h of heating at 40 °C. Once cooled, 1.0 mL of 12% (*w*/*v*) hydroxylamine hydrochloride was added and lightly swirled until a change in color was seen. A 1 mL aliquot of digestate was added to a 40 mL glass autosampler vial, along with 0.10 mL of 12% (*w*/*v*) tin (II) chloride and enough deionized water to fill the vial. Total mercury was determined by CVAFS with a Brooks Rand MERX-T analyzer. The concentrations of inorganic mercury (IHg) were estimated by subtracting the concentration of MeHg from THg in each liver. The concentrations of THg and MeHg in livers are reported on a dry weight (dry wt) basis. The accuracy and precision of MeHg and THg determinations were assessed by triplicate analyses of Dolt-2 (dogfish liver) and Tort-2 (lobster hepatopancrease) certified reference materials from the National Research Council of Canada, analytical and procedural blanks, replicate samples, and spiked samples.

The geometric mean concentration (±1 SD) of MeHg was 700.9 ± 22.5 µg/g (*n* = 7) in Dolt-2 and 151.6 ± 6.9 µg/g (*n* = 7) in Tort-2. Of the 21 individual analyses of Dolt-2, all but one were within the certified range of 640 µg/g to 746 µg/g. For Tort-2, 18 of 21 individual analyses were within the certified range of 139 µg/g to 165 µg/g. The precision (coefficient of variation) of samples analyzed in triplicate averaged 3.9%. Mean recoveries of MeHg from spiked samples were 101.6%.

The geometric mean concentration (±1 SD) of THg was 2229.4 ± 68.5.5 µg/g (*n* = 7) in Dolt-2 and 277.2 ± 6.2 µg/g (*n* = 7) in Tort-2. All 21 individual analyses were within the certified range for Dolt-2 (range 1800 µg/g to 2420 µg/g) and for Tort-2 (range 210 µg/g to 330 µg/g). The precision (coefficient of variation) of samples analyzed in triplicate averaged 3.8%. Mean recoveries of THg from spiked samples were 103%.

### 2.4. Selenium Determination

Fish livers were digested with HNO_3_ and analyzed for total Se by inductively coupled plasma mass spectrometry (ICP-MS), following U.S. EPA Method 6020a. Subsamples of freeze-dried, homogenized liver were accurately weighed (±0.001 g) into 68 mL polypropylene vials, to which 10 mL of high-purity HNO_3_ was added. The vials were covered with polypropylene watch glasses and heated at 95 °C for 6 h in a Class 100 fume hood, after which digestates were diluted to 50 mL with reagent-grade water (>18 MΩ-cm). Digestion batches included three procedural blanks, replicate digestions of DORM-4 (fish protein) and DOLT-2 (dogfish liver), triplicate digestions of >10% of samples, and replicate samples with known additions of Se made prior to sample digestion. Determinations of Se in samples were calibrated against aqueous standards, traceable to the U.S. National Institute of Standards and Technology. The mean (±1 SD) measured concentration of Se in DORM-4 was 3.69 ± 0.17 µg/g (*n* = 15), with all but one analysis within the certified range of 3.22 µg/g to 3.90 µg/g. All measurements of Se concentration in DOLT-2 were within the certified range of 5.57 µg/g to 6.55 µg/g and averaged 5.97 ± 0.12 µg/g (*n* = 21). Precision among triplicate digestates averaged 2.4 ± 2.8% relative standard deviation among 24 sets of matched samples, and recovery of known Se additions averaged 99 ± 6% (*n* = 72). The estimated limit of quantification was 0.11 µg/g, much less than measured Se concentrations in livers.

### 2.5. Statistical Analysis

Data were analyzed with IBM Statistics for Windows, version 22.0 (IBM Corp., Armonk, N.Y., USA). A non-parametric Kruskal–Wallis test was performed to determine differences among parks sampled, with a Dunn’s post hoc test used to determine differences in gene expression in the livers of northern pike from among the parks. Linear regressions were used to determine the relationship between Se, MeHg, THg, and between gene expression and %MeHg. Spearman pairwise correlations were conducted between normalized gene expression, Se, MeHg, and THg. Due to wedge shaped distributions, quantile regression analysis of genes relative to the molar concentrations of THg and Se was conducted using the program Blossom (version 2005.04.02) [41]. Gene expression outliers were determined if standard deviations were ±2 from the mean. Statistical significance was evaluated at *p* < 0.05.

## 3. Results

A total of 96 livers from northern pike were initially analyzed in this study. Standard deviations ± 2 from the mean of normalized gene expression were used to determine outliers. One fish exceeded this limit and was excluded from the results. Due to a limited amount of liver tissue for Se analysis, one additional fish was not included in the results, which resulted in a total of 94 livers analyzed for Se, Hg, and expression of *sod*, *gst*, and *mt*. Furthermore, 92 of 94 livers with detected expression of *cat* were used in analysis. 

**Figure 1:**
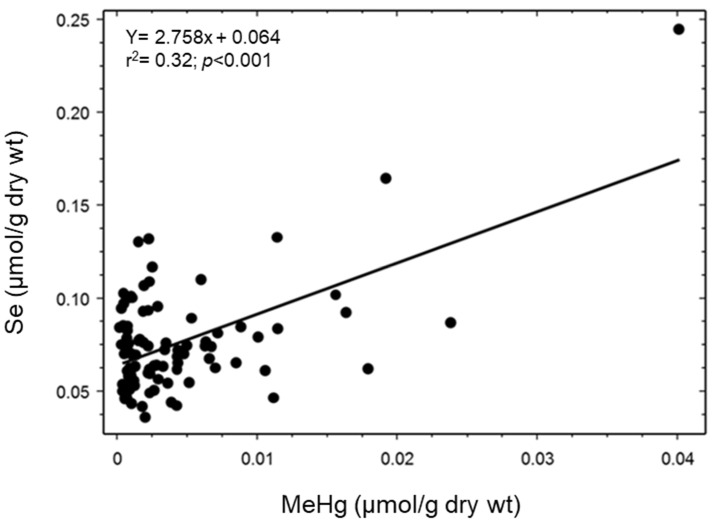
Relation between the concentrations of total selenium (Se) and methylmercury (MeHg) in livers of northern pike (F_1,92_ = 42.48; *p* < 0.001; r^2^ = 0.32; *n* = 94).

**Figure 2:**
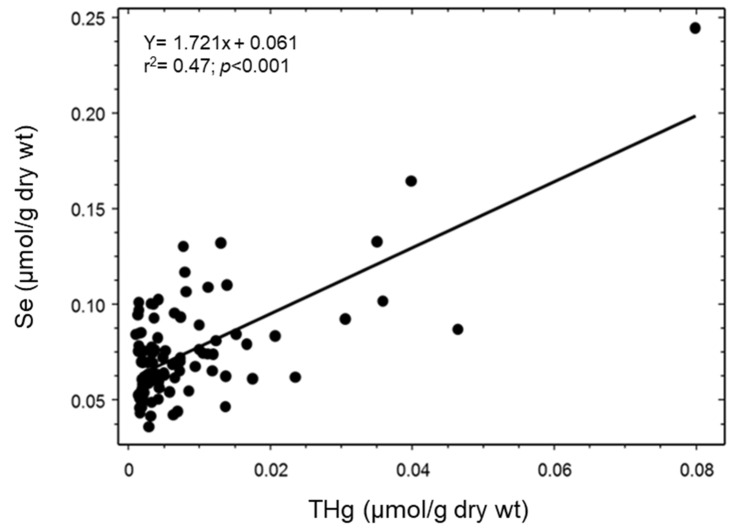
Relation between the concentrations of total selenium (Se) and total mercury (THg) in livers of northern pike (F_1,92_ = 80.10; *p* < 0.001; r^2^ = 0.47; *n* = 94).

There was a significant relation between the concentrations of MeHg and Se (F_1,92_ = 42.48; *p* < 0.001; r^2^ = 0.32; Figure 1) and THg and Se (F_1,92_ = 80.10; *p* < 0.001; r^2^ = 0.47; Figure 2) in livers of northern pike. 

Se concentrations ranged from 2.83 µg/g to 19.30 µg/g dry wt (mean = 5.95 ± 2.28), MeHg concentrations ranged from 0.04 µg/g to 8.65 µg/g dry wt (mean = 0.89 ± 1.27), and THg concentrations ranged from 0.20 µg/g to 16.03 µg/g dry wt (mean = 1.63 ± 2.30), with concentrations of Se in excess of Hg in all fish livers examined (Table 2). The highest concentrations of Se, MeHg, and THg were from livers of fish from Voyageurs National Park (VOYA), where there were significantly greater levels of MeHg (F_3,93_ = 12.29; *p* < 0.001; Table 2) and THg (F_3,93_ = 7.30; *p* < 0.001; Table 2) than those livers of fish from the other parks. 

Pairwise correlations determined that *sod*, *cat*, *mt*, and *gst* normalized mRNA expression were all positively correlated with each other, but unrelated to Se (µmol/g dry wt), MeHg (µmol/g dry wt), or THg (µmol/g dry wt) (Table 3). Metallothionein expression was significantly higher in livers of northern pike from Sleeping Bear Dunes National Lakeshore (SLBE) than those from VOYA (*p* = 0.031; average 49% higher) and Pictured Rocks National Lakeshore (PIRO) (*p* = 0.041; average 53% higher), while *sod*, *cat*, and *gst* expression in livers did not significantly differ among parks sampled (*p* > 0.05; Table 4). 

**Figure 3:**
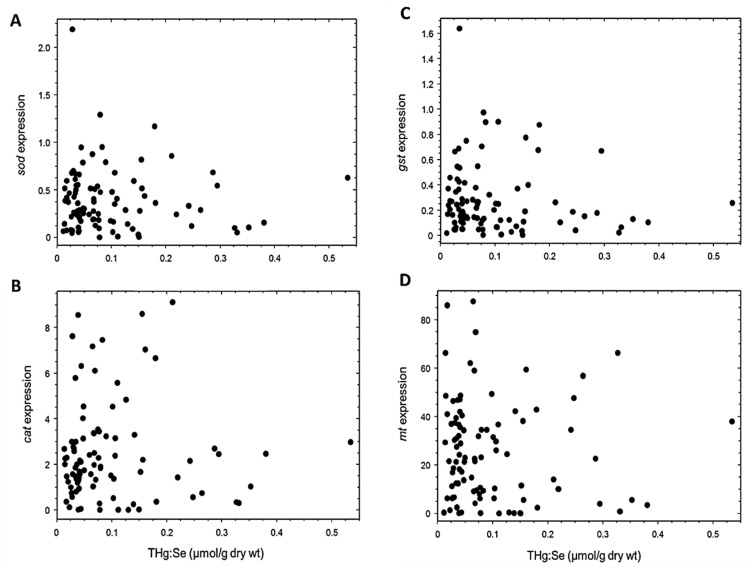
Quantile regression analysis between normalized (**A**) *sod* (τ 99; *p* = 0.481; n = 94), (**B**) *cat* (τ 99; *p* = 0.255; *n* = 92), (**C**) *gst* (τ 99; *p* = 0.524; *n* = 94), and (**D**) *mt* (τ 99; *p* = 0.741; *n* = 94) expression and molar ratio of total mercury to selenium (THg:Se) in livers of northern pike.

**Figure 4:**
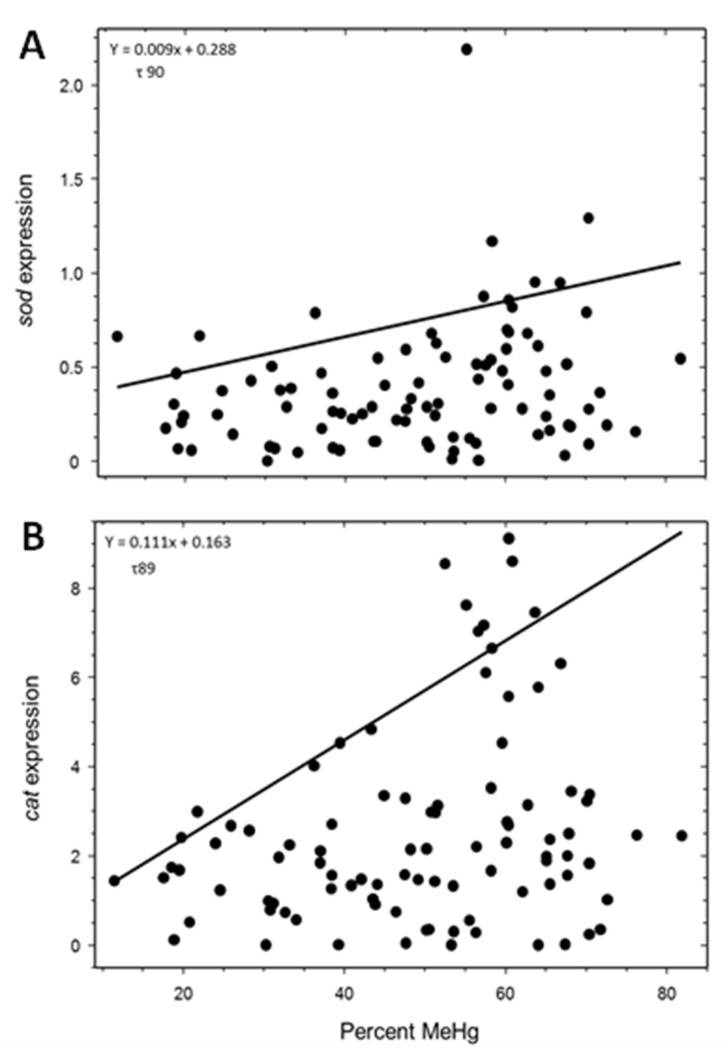
Quantile regression analysis between normalized (**A**) *sod* (τ 90; *p* = 0.013; *n* = 94) and (**B**) *cat* (τ 89; *p* = 0.041; *n* = 92) expression and percent total mercury as methylmercury (MeHg) in livers of northern pike.

Superoxide dismutase expression was not significantly related to the molar ratios of MeHg:Se (τ 75, *p* = 0.181), IHg:Se (τ 99, *p* = 0.480), or THg:Se (τ 99, *p* = 0.481; Figure 3A), although it was significantly upregulated with increasing percentages of THg as MeHg (τ 90, *p* = 0.013; Figure 4A). Catalase expression was not significantly altered by MeHg:Se (τ 99, *p* = 0.215), IHg:Se (τ 99, *p* = 0.441), or THg:Se (τ 99, *p* = 0.255; Figure 3B) molar ratios, although it was significantly upregulated with increasing percentages of THg as MeHg (τ 89, *p* = 0.041; Figure 4B). Expression of *gst* was not significantly changed with increasing MeHg:Se (τ 99, *p* = 0.483), IHg:Se (τ 80, *p* = 0.151), or THg:Se (τ 99, *p* = 0.524; Figure 3C) molar ratios, and was not significantly altered with an increasing percentage of MeHg (τ 95, *p* = 0.141). 

Metallothionein expression did not change significantly with increasing MeHg:Se (τ 80, *p* = 0.723), IHg:Se (τ 90, *p* = 0.183), or THg:Se (τ 99, *p* = 0.741; Figure 3) molar ratios, and was not significantly altered with an increasing percentage of MeHg (τ 95, *p* = 0.268). Metallothionein expression was significantly related to Se (µmol/g dry wt) (τ 95, *p* = 0.041; Table 3). Conversely, *sod* (τ 85, *p* = 0.065), *cat* (τ 85, *p* = 0.600), and *gst* expression (τ 90, *p* = 0.203) were not significantly related to Se (µmol/g dry wt) (Table 3). 

## 4. Discussion

Most studies that use biomarkers to assess the toxicity of Hg in organisms have been primarily lab-based [20,42,43,44,45,46,47], with only limited field application [21,48,49]. Dietary Se is an essential nutrient that is not always administered at environmentally relevant concentrations during toxicity studies in the lab setting. Due to the protective role Se has in reducing effects of Hg toxicity [25,27,29,50], a molar ratio between Hg and Se has been suggested to be a better predictor of Hg toxicity than reporting Hg alone [23,28,51,52,53,54,55,56,57,58]. The protective role of Se to Hg toxicity, however, remains uncertain, particularly due to a lack of studies related to the mechanistic understanding of binding capacity and bioavailability in fish [36,59]. Limitations in the relationship of Hg:Se molar ratios between freshwater and marine species further increases uncertainty, particularly as it relates to fish health.

Selenium concentrations significantly increased relative to THg concentrations in all livers examined, and have been similarly shown to be positively related to Hg concentrations [60,61]. Selenium concentrations were in abundance of Hg in all fish livers examined, with THg concentrations in northern pike livers similar to those collected from Isle Royale (range 0.048 µg/g to 3.074 µg/g wet wt) by Drevnick et al. (2008) [22]. When moles of Se are less than Hg, the sequestration of Se by Hg reduces the available Se needed for proper selenoenzyme synthesis [56], which could elicit other stress-induced systems to remove Hg or catalyze free radicals generated through oxidative stress. However, although Se had a positive relationship with MeHg in livers, there was not a significant correlation. The mechanistic relationship between Se and MeHg has been largely based on mammalian models [36], and additional study is needed to understand how the dysregulation of gene expression relates to Hg:Se molar ratios in fish.

Superoxide dismutase is among the first genes to defend against reactive oxygen species, specifically the superoxide radical (O_2_^−^). Superoxide dismutase catalyzes the conversion of O_2_^−^ into H_2_O_2_, which can be converted into non-toxic components. Ji et al. (2006) [62] fed rats a diet of rice containing Hg and Se for 7, 20, 30, and 90 days, with Hg and Se doses increasing relative to exposure time. After day 30, sod activity significantly decreased in rat livers relative to that in control groups, regardless of Hg and Se exposure. This suggests that prolonged exposure to Hg and Se-containing diets could suppress the activity of certain enzymes responsible for cellular response to oxidative stress. To assess the potential effects of Hg to humans, Grotto et al. (2011) [63] fed groups of rats either a diet of conventional rat food (control) or fish contaminated with MeHg. Livers from rats fed fish contaminated with MeHg had a mean Hg concentration of 0.870 ± 0.030 µg/g and a mean Se concentration of 1.9 ± 0.2 µg/g. Similar to northern pike livers, the mean molar Se concentrations in the livers of the rats fed MeHg-contaminated diets were greater than mean molar Hg concentrations. No variation in sod or cat activity were seen between the contaminated fish diet and the control diet, suggesting that Se could have a protective effect against Hg toxicity when in a higher molar concentration than Hg [63]. In contrast, Gonzalez et al. (2005) [42] found that feeding zebrafish a diet with 5 µg and 13.5 µg Hg g^−1^ for 21 days resulted in a 3-fold and 12-fold increase in the expression of *sod*. The expression of *sod* was highest in the liver, suggesting that the basal levels of *sod* were great enough to protect the cells from oxidative damage due to MeHg exposure. 

Similar to Gonzalez et al. (2005) [42], we also observed a similar response in *sod* expression in livers of northern pike. The expression of *sod* and *cat* were expressed at low levels of MeHg as a percentage of THg (%MeHg), which could be representative of a baseline level of enzyme expression. Expression significantly increased with a greater %MeHg. Examining the %MeHg could prove beneficial due to Se having a higher affinity for IHg than MeHg [64], allowing MeHg to potentially induce oxidative stress. There was, however, no significant difference in *cat* or *sod* expression when considering Hg and Se individually or Hg:Se molar ratios. Similarly, when exposed to a diet containing MeHg, *cat* expression did decrease when exposed to diets containing MeHg and Se [65]. This suggests that a threshold exists between MeHg and Se that would prevent other antioxidant defenses from being expressed when Se was in excess of Hg. Graves et al. (2017) [49] examined populations of yellow perch (*Perca flavescens*) chronically exposed to a gradient of MeHg concentrations in lakes of Kejimkujik National Park. They reported that *cat* was the only gene that was significantly downregulated in the brains of perch, suggesting oxidative stress was due to Hg contamination. It is possible that other environmental factors, such as Se abundance, could affect these primary response genes associated with oxidative responses and have a role in gene expression. Because Hg is an irreversible inhibitor of selenium-containing proteins [23], selenium-containing enzymes could be a better predictor for Hg toxicity than non-selenium-containing proteins when Se is in a greater molar concentration than Hg and provide more information about the mechanism of interaction between Hg and Se. 

Although Se is an efficient scavenger and has a high binding affinity for Hg, gsh and mt have an important role in reducing Hg and oxidative damage associated with Hg toxicity. The gsh system is important in removing ROS generated by oxidative stress as well as converting xenobiotics, such as Hg, into more soluble forms to be excreted. Glutathione s-transferase is an important enzyme in activating sulfhydryl groups on gsh and conjugating Hg to gsh [66]. However, *gst* expression in livers of northern pike was not significantly correlated with Se, MeHg, THg, or Hg:Se molar ratios. A similar response was seen in walleye (*Sander vitreus*) from boreal forest lakes in Canada [21]. The activity of gst was not related to THg or MeHg concentrations in the livers of walleye but decreased significantly in livers of yellow perch with increasing MeHg. There was also a great variability in gst activity between the two species and among the lakes sampled [21]. This could suggest that differences in the response of enzyme activity are dependent on fish species and metal concentrations [67]. 

Metallothionein is an important protein that sequesters heavy metals and removes them from cells. The expression of *mt* is induced by Hg [46,68], although mt only binds and removes IHg, not MeHg [69]. The expression of *mt* was not significantly altered with increasing concentrations of Hg but was significantly upregulated with increasing Se concentrations. A similar response was seen in Atlantic cod, where *mt* expression was unrelated to MeHg exposure but increased with exposure to Se-enriched food [65]. The expression of *mt* in livers of northern pike was also not significantly related to Hg:Se molar ratios, which may be attributed to a greater molar concentration of Se than Hg and suggests a higher binding affinity of Hg to Se than to *mt*. It is possible that Se had a protective effect against Hg toxicity due to Hg:Se ratios < 1 in all fish or that the baseline expression of the oxidative stress genes was already elevated due to prolonged exposure to Hg. The adaptive response of oxidative stress genes has been reported previously with metals, metalloids, and additional contaminants of emerging concern [70,71] and in field-collected fish [72,73].

## 5. Conclusions

As more becomes known about the implication of Se in the diet of Hg-containing organisms with its ability to ameliorate Hg toxicity, using Hg:Se molar ratios when addressing issues with Hg may be important to consider. Incorporating biomarkers containing Se may provide a better means of understanding the interaction between Hg and Se, and the role Hg has in the sequestration of Se, particularly when molar concentrations of Se are greater than Hg. Furthermore, the baseline expression of oxidative stress genes may be greater in areas where northern pike are subjected to prolonged exposure of Hg from atmospheric sources. With a narrow range between Se essentiality and toxicity, further research is needed to understand how these concentrations not only effect different organisms, but how the addition of Hg alters that specified range. Gaining a clearer representation of what those concentrations are, and the interaction they have with Hg, will aid in better assessment of using ecologically relevant concentrations of Se in lab-based studies and give insight into adverse effects of Hg contamination in Se-poor geographic areas. 

## Figures and Tables

**Table 1:** Genes and primers used in qPCR analysis of livers in northern pike.

Gene	Direction	Primer (5′–3′)	Accession Number
Glutathione s-transferase	Forward	GACTTCCCAGAATGGATGAAGG	BT07989.1
	Reverse	TGACTGAAACAGGACCAAATCA	
Metallothionein	Forward	CTGGATCTTGCAACTGTGGT	X59392.1
	Reverse	CTTGCTGCAACCAGAAGGA	
Superoxide dismutase	Forward	CGCAGAGGACAAGTACAAAGA	BT079033.1
	Reverse	GATGTGGCCTCCTCCATTAAA	
Catalase	Forward	GTGGGAAAGACCACACCTATC	BT045615.1
	Reverse	GTTTCCCTCGTCAGTGTAGAAC	
Ubiquitin	Forward	GCCTTTCCTACCTGACAGTATTC	BT079424.1
	Reverse	AAAGTCAACGCTCCATCTCC	

**Table 2:** Characteristics (mean ± SD) of northern pike collected from Isle Royale National Park (ISRO), Pictured Rocks National Lakeshore (PIRO), Sleeping Bear Dunes National Lakeshore (SLBE), and Voyageurs National Park (VOYA).

Park	n	Wet Weight (kg)	Total Length (mm)	Se (µg/g Dry Weight)	MeHg (µg/g Dry Weight)	THg (µg/g Dry Weight)	THg:Se Molar Ratio
ISRO	25	1.24 ± 0.49	572 ± 87	6.345 ± 2.299	0.311 ± 0.473	1.034 ± 1.414	0.064 ± 0.0496
PIRO	26	1.66 ± 1.27	586 ± 147	4.935 ± 1.176	0.490 ± 0.366	0.852 ± 0.614	0.068 ± 0.050
SLBE	8	0.77 ± 0.27	493 ± 53	6.156 ± 1.715	0.196 ± 0.110	0.360 ± 0.113	0.023 ± 0.022
VOYA	35	0.86 ± 0.42	527 ± 94	6.371 ± 2.787	0.177 ± 1.692	2.922 ± 3.146	0.181 ± 0.117

**Table 3:** Spearman correlation for gene expression and selenium (Se), methylmercury (MeHg), and total mercury (THg) in northern pike. The *p*-value of the test is presented in parentheses.

	*sod* Expression	*cat* Expression	*mt* Expression	*gst* Expression	Se (µmol/g dry wt)	MeHg (µmol/g dry wt)	THg (µmol/g dry wt)
*sod* expression	1						
*cat* expression	0.698 (0.000)	1					
*mt* expression	0.311 (0.002)	0.377 (0.000)	1				
*gst* expression	0.702 (0.000)	0.503 (0.000)	0.229 (0.026)	1			
Se (µmol/g dry wt)	−0.157 (0.130)	−0.092 (0.385)	0.197 (0.057)	−0.122 (0.240)	1		
MeHg (µmol/g dry wt)	0.021 (0.839)	0.114 (0.279)	−0.095 (0.364)	−0.102 (0.329)	0.178 (0.085)	1	
THg (µmol/g dry wt)	−0.057 (0.586)	0.040 (0.702)	−0.065 (0.534)	−0.141 (0.175)	0.347 (0.001)	0.919 (0.000)	1

Superoxide dismutase = *sod*; catalase = *cat*; metallothionein = *mt*; glutathione s-transferase = *gst.*

**Table 4:** Range and mean (±SD) values of normalized gene expression in northern pike collected from Isle Royale National Park (ISRO), Pictured Rocks National Lakeshore (PIRO), Sleeping Bear Dunes National Lakeshore (SLBE), and Voyageurs National Park (VOYA).

	Park			
Gene Expressed	ISRO	PIRO	SLBE	VOYA
*sod*	0.0003–0.786 n = 25 (0.281 ± 0.205)	0.057–0.950 n = 26 (0.450 ± 0.259)	0.074–2.189 n = 8 (0.671–0.716)	0.004–1.168 n = 35 (0.352 ± 0.279)
*cat*	0.0006–4.015 n = 24 (1.480 ± 0.925)	0.006–8.598 n = 26 (2.896 ± 2.226)	0.354–7.616 n = 8 (2.430 ± 2.208)	0.002–9.109 n = 34 (2.898 ± 2.535)
*mt*	0.142–61.958 n = 25 (24.463 ± 17.326)	0.133–74.851 n = 26 (22.279 ± 18.595)	23.095–85.840 n = 8 (47.120 ± 20.385)	0.036–87.455 n = 35 (24.111 ± 21.974)
*gst*	0.003–1.637 n = 25 (0.315 ± 0.348)	0.048–0.897 n = 26 (0.261 ± 0.222)	0.169–0.973 n = 8 (0.367 ± 0.266)	0.004–0.899 n = 35 (0.233 ± 0.235)

Superoxide dismutase = *sod*; catalase = *cat*; metallothionein = *mt*; glutathione s-transferase = *gst.*

## Data Availability

Not applicable.
